# Small Successes Make Big Wins: A Retrospective Case Study towards Community Engagement of Low-SES Families

**DOI:** 10.3390/ijerph17020612

**Published:** 2020-01-18

**Authors:** Lotte Prevo, Stef Kremers, Maria Jansen

**Affiliations:** 1 NUTRIM, Department of Health Promotion, Maastricht University, 6229 HA Maastricht, The Netherlands; s.kremers@maastrichtuniversity.nl; 2 CAPHRI, Department of Health Services Research, Maastricht University, 6229 HA Maastricht, The Netherlands; maria.jansen@ggdzl.nl; 3 Academic Collaborative Center for Public Health, Public Health Service Southern Limburg, 6411 TE Heerlen, The Netherlands

**Keywords:** community engagement, low-SES families, self-resilience, health promotion participation

## Abstract

In health-promoting interventions, a main difficulty is that low socioeconomic status (SES) groups especially seem to experience barriers to participation. To overcome this barrier, the current study focused on the success factors and obstacles in the process of supporting low-SES families in becoming partners, while carrying out small-scale activities based on their needs. A retrospective case study design was used to construct a timeline of activities organized by and together with low-SES families based on mainly qualitative data. Next, key events were grouped into the four attributes of the resilience activation framework: human, social, political, and economic capital. The following key lessons were defined: professionals should let go of work routines and accommodate the talents of the families, start doing, strive for small successes; create a functional social network surrounding the families, maintaining professional support over time as back-up; and create collaborative governance to build upon accessibility, transparency and trust among the low-SES families. Continuous and flexible ‘navigating the middle’ between bottom-up and top-down approaches was seen as vital in the partnership process between low-SES families and local professional partners. Constant feedback loops made the evaluation points clear, which supported both families and professionals to enhance their partnership.

## 1. Introduction

The unequal distribution of health between individuals from low socioeconomic status (SES) groups and middle-to-high SES groups, so-called health disparities, remains a major challenge in health promotion [1,2]. Globally, a higher morbidity and mortality have been reported among socially disadvantaged groups [3]. Besides physical health outcomes, low-SES groups also score worse on mental and social health outcomes. These groups often show lifestyle behaviors that help them cope with their stressful life situation, while at the same time exposing themselves to negative health effects and making their situation worse [4]. A positive mediating effect on the relation between SES and overall health was found to be a person’s lifestyle [5]. Health improves through the lifestyle choices that individuals make, e.g., engaging in physical activity, having social interactions and enhancing stress management skills [5]. Especially for low-SES groups, the benefits of enhancing their physical, mental and/or social health are considered to be a key step to closing the health gap [4]. 

The main difficulty is that low-SES groups especially seem to experience barriers in terms of participating in activities to support their general health [6,7]. Due to the daily issues they perceive, they carry a high mental burden, depleting their cognitive energy to take part in health-promoting programs and act upon health-related goals [8,9]. Despite the many well-intended health promotion programs, the broader living situation and the needs of the low-SES groups are often inadequately taken into account, and therefore these programs often show low participation by members of the low-SES group and high levels of drop out [2]. It is still a challenge to expend more effort to include low-SES groups in the community engagement process of developing, implementing, and evaluating health promotion programs from the very beginning [10]. The higher level of involvement of, and partnership with, the low-SES groups implies a central focus on their needs, and balancing between top-down knowledge and processes introduced by professionals and bottom-up perceived needs and work processes of low-SES groups [11]. 

With the idea of making low-SES families an equal partner and involving them in health-promoting activities from the beginning, the Vaals Meets project was developed. Half a year before the project started, eight low-SES families took part in a photovoice study to define their needs [12]. They were asked to make pictures of daily issues and opportunities they perceived to improve their quality of life. The photovoice study led to four themes relevant to low-SES families: 1. meeting each other, 2. helping each other, 3. feeling safe, and 4. being mobile. The themes supported the desire of the families to be independent and self-resilient. It was interesting that the themes were not specifically focused on health but revealed the importance of first tackling their basic needs before opportunities could arise to enhance their health status [12]. These four themes formed the basis of Vaals Meets, in which families were supported with organizing small-scale activities and cooperating with formal and informal community partners, with the aim to strengthen their feeling of independence and self-resilience. These small-scale activities focused on upstream determinants of health, such as exploring your own talents or enhancing social support. Over the long term, they are expected to have a trickle-down effect on the families enacting them, encouraging them to accomplish greater steps in independence and self-resilience and eventually reach a better health status [13].

This paper focuses on the process to support low-SES families in becoming equal and active partners in carrying out small-scale activities based on their needs. The overall aim was to define success factors and obstacles that were encountered while partnering with the low-SES families in the organization of small-scale activities. Consequently, we aimed to synthesize key principles in the process to strengthen the position of low-SES families in health promotion development.

## 2. Materials and Methods 

### 2.1. Study Design

Through participatory action research (PAR), all activities carried out by low-SES families were carefully monitored to create a better understanding of their reactions, perceptions and feelings [14,15]. Feedback loops were created and stimulated by the participatory researcher to accelerate processes in the system. We used a retrospective case study design. A timeline of activities reconstructed by the researcher, the families and the two key stakeholders (activation broker and policy maker) provided insight into the events and variables that changed over time. Each activity included in the timeline was accompanied by a core description of the situation. By using a relativism paradigm, we placed a focus on describing, exploring and giving meaning to the events that happened. This helped us to understand key principles in the process of engaging low-SES families [16]. An inductive process was used to compose the timeline. As a next step, an abductive process [17] was used to synthesize the key lessons learned by using a theory that fitted well to the events described on the timeline.

### 2.2. Study Setting

From April 2016 to February 2018, the study took place in the municipality of Vaals, a small town located in the southernmost part of the Netherlands, near the border with Germany and Belgium. The municipality has approximately 10,000 citizens, of mainly Dutch origin, and a moderate to low SES. Consequently, its citizens have a poorer health status, a shorter life expectancy, more mental health issues, and an unhealthy lifestyle compared with the average Dutch population [18]. Accompanying issues such as living in poverty and experiencing stress were also frequently described [18,19]. The Public Health Service Southern Limburg, Maastricht University, and the municipality of Vaals, provided support for this study. We performed the study in accordance with the Code of Conduct for Health Research of the Dutch Federation of Biomedical Scientific Societies.

### 2.3. The Resilience Activation Framework

In the process of analyzing the events on the timeline, the resilience activation framework (RAF) [20] was used to categorize the lessons learned. The RAF approached resilience as a process in which the capacity to withstand or recover from disturbances that threaten the existing quality of life is created on an individual and/or community level. Within RAF, four attributes on the individual and community levels explained the presence of capital that remained underutilized, making resilience to flourish impossible: human, economic, social, and political capital. Moderate-to-high SES groups were expected to be able to use their capital, e.g., good health and a positive mindset (human capital), savings (economic capital), a strong social network (social capital), and access to people in leadership positions (political capital). Low-SES groups were expected to have lower capacity levels of capital, making it harder to gain resilience. In the analysis phase of our study, we focused on explaining and understanding changes with these attributes. 

### 2.4. Recruitment and Study Participants

In the process of recruiting the low-SES families, an intermediary, a so-called activation broker, played a central role. The broker was familiar to the local citizens and had good contacts with professional partners. The personal contacts she had made the recruitment process successful for the Photovoice study (July 2015–January 2016). From the ten family members who participated in this needs assessment phase, eight members decided to continue. By using a snowball sampling technique, participants fostered recruitment of more families. To create engagement from the community as a whole, formal and informal partners, including other citizen groups, were welcomed to work together with the families in creating activities focused on the earlier defined needs. From the 220 low-SES families in Vaals [21], twelve low-SES families with various backgrounds, e.g., single-parent families, complete families, and families from Dutch and other origins, actively participated during the study period. More families, approximately 40, were mainly engaged as visitors during the activities organized by these families.

### 2.5. Community Engagement Approach

The involvement and facilitation of low-SES families during the planning phase and organization of their own activities was a central aspect of the community engagement approach. After defining the most important needs and accompanying themes with the families, the professionals (activation broker, policy maker, and researcher) and the families decided that it was time to become active. Workgroups were created by the professionals in which the families started to prepare, discuss, organize, and evaluate small-scale activities, with their support. Besides these professionals and the low-SES families, connections with other formal and informal partners were sought in the community with the aim of creating a collaborative partnership. A policy maker at the tactical level from the municipality of Vaals played an important role at the nexus of local policy, the broker, the researcher, and the family’s needs to actually realize activities. This achieved a professional cooperation between policy, practice, and research. The small-scale activities, accompanying processes, and results of the workgroups were monitored and evaluated by the researcher and used as input for new small-scale activities. 

### 2.6. Data Collection, Instrument, and Measurements

This study mainly involved the use of different types of qualitative research instruments to gather data. First, during the multiple workgroup sessions organized with the families, optional activities, minutes of the discussion, and agreements made by the researcher, the broker, and the families were discussed. The researcher took notes about the group process. Second, during the actual small-scale activities organized by the families, the researcher undertook observations and made notes as well. These observations and notes mainly focused on success factors and learning points to enhance self-resilience among the families, but also included some quantitative outcomes such as number of visitors. All activities were evaluated during the meetings with the families, and successes were mentioned (and celebrated). Finally, twice in the research period a focus group interview was held in which actively involved low-SES families at that point were invited and asked to describe their experiences with the approach, what they considered good, where there was room for improvement, and what goals they wanted to achieve in the upcoming period. After this interview, the families, broker, and researcher had dinner together. 

### 2.7. Data processing and Analysis 

All data gathered from observations, minutes of meetings, process notes and observation of meetings, events, and focus group interviews were organized with Nvivo software to create the timeline with activities [22]. After creating the complete timeline of all described activities, the researcher (LP) worked together with the project team members (MW and JM) who were closely involved in the PAR research trajectory to complete the timeline and structure the timeline activities into attributes within the RAF. By creating a complete timeline and reconstructing essential supportive and hindering attributes in the participatory process, we synthesized key lessons. 

## 3. Results

**Figure 1:**
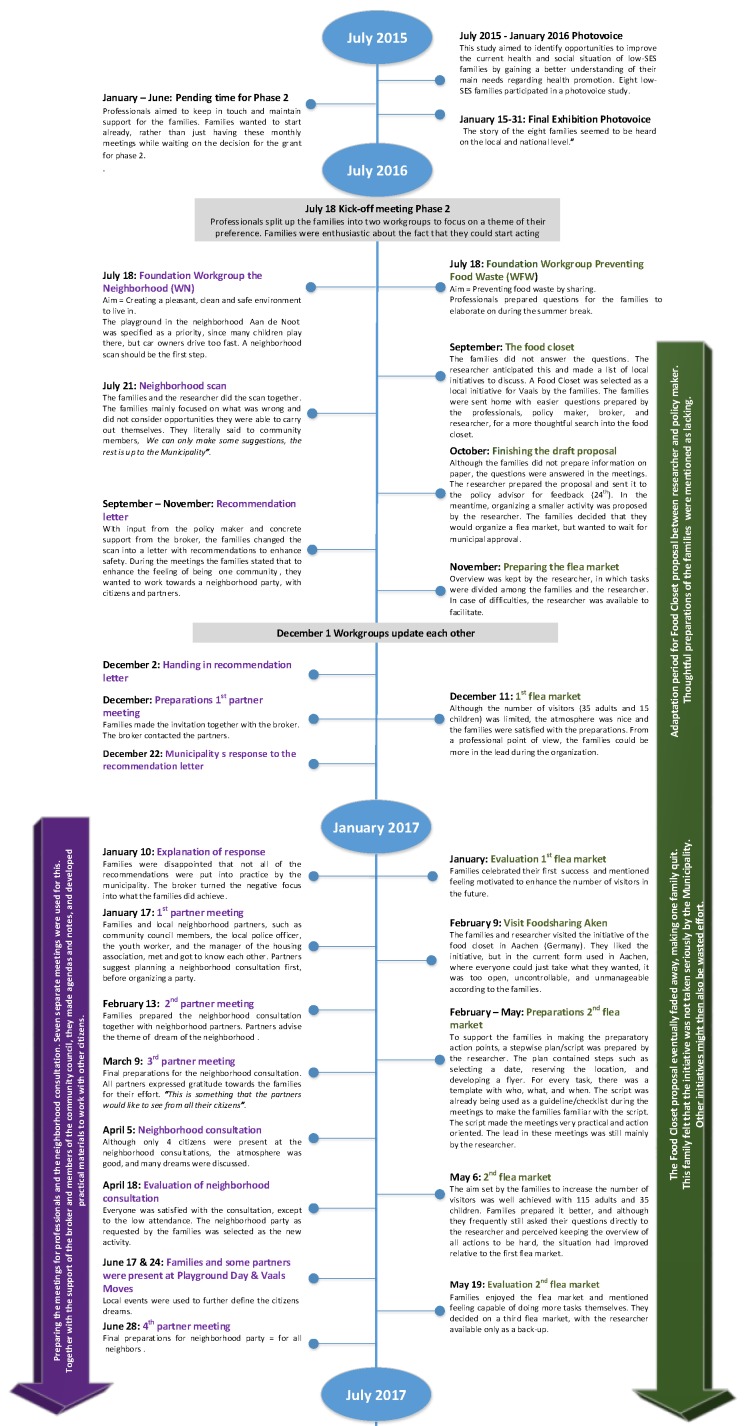

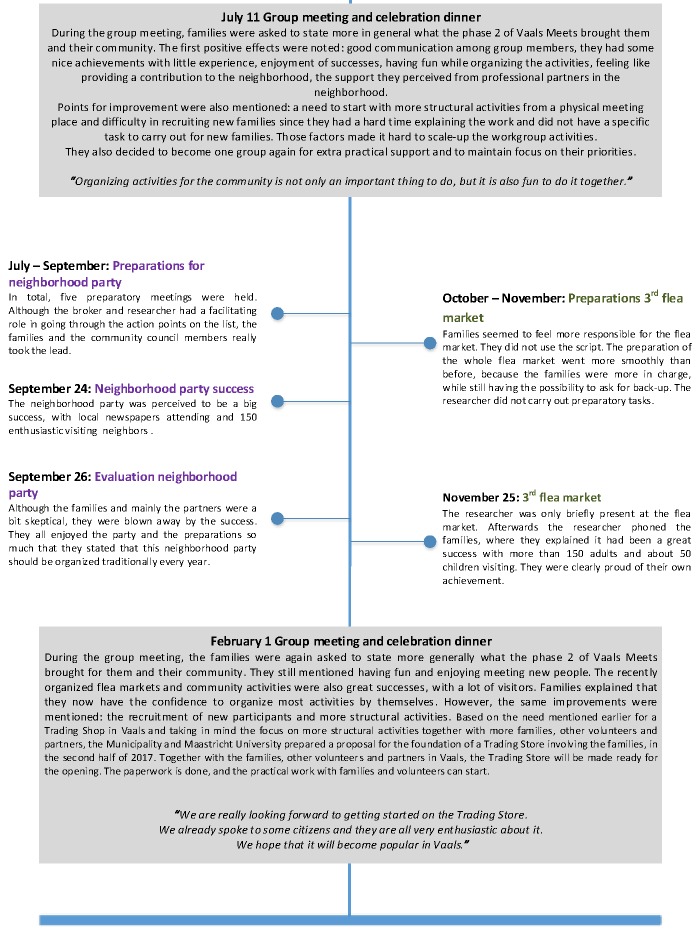

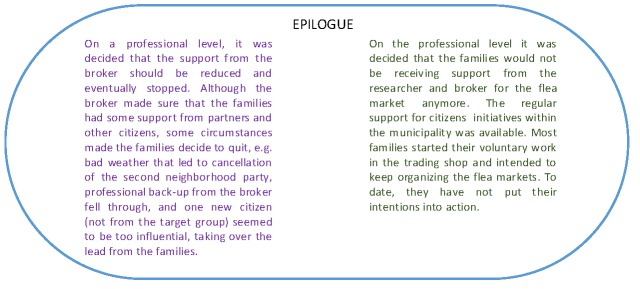
Timeline.

During the first year from July 2016 to July 2017, the families received relatively intense support from the broker and/or researcher (Figure 1). During the final half-year (July 2017–February 2018), families more often took the lead independently. This indicated a change from a top-down approach by the professionals to more bottom-up input from the families. The constant feedback loops created enhancements in the human, social, and political capital, with the largest growth being observed in human capital. Changes over time in all types of community capital will be discussed separately below, concluding with key lessons learned that implied a navigation between top-down and bottom-up approaches (Table 1).

### 3.1. Human Capital

To enhance the human capital, the broker’s and researcher’s training support for low-SES families offered them the skills they needed to organize small-scale activities. Professionals, the broker and researcher, first had to assist and encourage families to take the lead in their activities. Gradually, this top-down support was only needed as a back-up, and therefore adjusted. The families contributed from bottom-up to the community’s human capital by providing their ‘expert knowledge’ to the activities, since they best knew what their community needed. A transition from top-down to more bottom-up became visible. 

It took some time for the families to become an actively involved partner and to successfully realize small-scale activities. Time was needed to bridge the gap between the professionals’ partnership approach and that of the families in making preparations and organizing small-scale activities. Professionals used a ‘talking-approach’ whereas the families used a ‘doing-approach’. A ‘brilliant failure’ after many ‘talking’ sessions was the idea of the food closet (see Timeline) that never materialized and faded out. According to the municipality, the proposal was never perceived as sufficiently and thoroughly explained in order to start implementation. 

Realizing small-scale activities showed families about personal growth throughout the preparations and organization. They first focused on the things that might go wrong, were more insistent on actually starting with the activities themselves, and waited for approval from the broker/researcher. It is likely that professionals reinforced this due to their actions at the beginning. For example, the researcher kept sending families of the Preventing Food Waste Workgroup back home with new questions to answer, and during the first meeting with community partners (i.e., community council members, the local police officer, and the youth worker), the partners decided that the families should start with a neighborhood consultation, before organizing a neighborhood party. 

During the final half-year, the families acted as really involved partners, showing the capabilities to lead their own activities. Successful small-scale activities were considered beneficial to a more positive mindset. Pessimism seemed to have changed into positivism. The families saw opportunities for themselves and their community and started to work more independently, while professionals noticed the growth in their capabilities. Difficulties were also encountered, e.g., dealing with some setbacks and the relatively long policy procedure for the food closet proposal, which made expectation management from the broker and researcher important to keep the families going. Eventually, the families felt able to carry out this type of activity themselves and were willing to be part of a more consistent initiative. Concerning the professionals, we saw the necessity to have a flexible and openminded attitude and focus directly on the preferred activities of the families, while providing support and facilitation, as an equal partner. 

### 3.2. Social Capital

As a second form of capital, social capital, seemed to grow during the study period. The workgroups created a new social network for the families, by working towards a common goal. Recruiting new families and community partners remained the major difficulty. The broker played a necessary role in recruiting more families, while also including other citizens and partners in the community to create a broader partnership within the workgroups. Although initiated in a top-down manner, the broker’s broad network and contacts enabled her to strengthen the families’ network. This created a supportive network surrounding the families to fall back on. Due to the equal partnership and leading position of the families created in the workgroups, their status on the social hierarchy seemed to increase. The families were proud of their work and the realized partnerships. All partners and visitors to their small-scale activities complimented the families. 

Most families were not familiar with each other before the project. During the photovoice phase, they quickly became companions due to their mutual understanding of each other’s situations. For the researcher and the broker, it was important to create trust to start partnering as well. The broker was already familiar with the families and seemed to gain the status of ‘coworker’ faster than the researcher. The Neighborhood Workgroup with the support of the broker had greater resilience steps at the start, compared to the researcher’s group (Preventing Food Waste Workgroup). The perceived social support that the families received from their partnering families, the researcher, the broker, other citizens, and neighborhood partners was described as pleasant and helpful by the families. Having fun and enjoyment was important to the families, as did celebrating successes together. Although the families made great progress in strengthening social partnerships, the back-up, mostly from professionals, to facilitate processes (e.g., assist in the application for the permits for the flea market and neighborhood party) was found to be important. Maintained functional support was important for the families to share successes, but also to receive support when issues were encountered over time. 

### 3.3. Political Capital

The third form of capital, political capital, seemed to be enhanced by the close involvement of the policy maker that mostly facilitated the small-scale activities by the families. For example, during the neighborhood scan, the recommendation letter written by the families was already provided with feedback by the policy maker. Most suggestions made by the families were facilitated or turned into actions by the municipality. Conversely, the government also caused delay, due to the lengthy political process. The government can actually make or break a societal initiative. Collaborative governance was found to be important, as the government, the families, and other involved citizens and partners had to communicate well and work together to achieve successes that they could not achieve on their own. In this strategy the whole societal network of a municipality can be included, with the government being just one of the partners within the network. 

The close connection that the families had with the person in a ‘leadership’ position at the municipality was mainly helpful. The families appreciated the presence of the policy maker during the partner meetings to explain the input from the municipality. It showed the engagement and involvement of the municipality to the families, something they seemed to miss before. Although the policy maker was very outreaching and accessible for all families, the moment when the municipality had to say ‘no’, the families seemed to fall back in an ‘I told you so’ attitude. The municipality was depicted as non-collaborative, ‘controlling’, or ‘holding back progress’. A high level of involvement, openness, and transparency of the responsible policy maker, but also the ability to communicate about what is realistic, was seen as being important to maintain the families’ trust. 

### 3.4. Economic Capital

Finally, financial resources were made available for the small-scale activities from external funding of the Vaals Meets project. The families first intended to make some illogical choices according to the professionals, e.g., wanting to buy electric devices such as a coffee machine and kettle for the flea market. Subsequently, a focus was placed on sustainable and affordable options, such as borrowing the electronic devices, something the families seemed to be good at.

Before starting in Vaals Meets, most families had already received individual guidance from professionals at the community level, e.g., via social work, the credit bank, or the activity broker. The partnership seemed to support the families to become even more active in the neighborhood. Some families found employment during the study period. This had a positive influence on their income. It is hard to state the influence of Vaals Meets on the economic capital achievements of the families, because the nature of the research design did not focus on finding causal relations. However, we do expect that the equal partnership and the small-scale successes contributed to some extent to the ability of the families to find a job again.

## 4. Discussion

The current study aimed to define key lessons learned in the partnering process between professionals, e.g., the involved broker and researcher, and low-SES families. The organization of small-scale activities was used as a means to strengthen the position of low-SES families and create an equal partnership. With all the key lessons learned, a flexible and adaptive ‘navigating the middle’ route was seen as vital: governance needs to navigate between bottom-up processes with families in the lead and top-down approaches (led by the expertise of e.g., the broker and researcher) [23]. During the project period, a transition became visible that characterized families in becoming more self-resilient and independent, while the professionals, broker and researcher, had to be flexible to stay supportive and facilitating. Constant feedback loops, primarily created by the participatory researcher, produced insight into the process of partnering together, learning together, but also failing together. Over time, this supported both the families and the professionals to enhance their partnership, and a gradual growth became visible in the competences, autonomy, and relatedness of the families to take the lead [24]. At the final half-year, the empowered families clearly had their leading position and explained having the confidence to organize activities mainly by themselves. 

In terms of the collaboration between the low-SES families and the professionals, namely the broker and the researcher, a particular learning curve was evident. Although the professionals were in place to support and facilitate the partnership with the families by adding beneficial expert knowledge and skills to fulfill their mission [25], it seemed that the routinely used, professional, top-down approaches were not beneficial to discover the families’ talents. The first period mainly focused on meetings to talk and discuss topics, write recommendations and proposals to the municipality, and organize partner meetings. The families were not used to this way of working, talking, and writing. Gradually, they became more involved and learned from the activities [25,26]. An ongoing transition towards an equal partnership, empowerment, growth in optimism and self-resilience appeared when the families were able to start doing, e.g., planning and organizing activities, and to see and celebrate their results [26]. The utilization of the families’ strengths, with a focus on partnering with families based on their passions while giving them the actual recognition for their input, was seen as important [27]. It may have created a growth mindset among the families. The idea that their abilities are not fixed and can be developed may have had an enhancing effect on their self-resilience and partnership [28]. Although all stakeholders may have struggled to navigate the middle at the start, their human capital flourished during the study period due to the enhanced levels of participation.

The social networks of the families grew. Within this process, the broker ensured that the social support of the families was not only structural, when significant others were available, but also functional, when the families actually perceived the support as helpful and mutual [29]. The broker’s broad network made it relatively easy to connect the families with neighborhood partners and other citizens to create a stronger network to fall back upon. The broker was a trusted and familiar face to the families, since some of them already received individual support and because she had been active in the municipality for four years. Where the researcher struggled at the start to gain this level of trust, the broker succeeded in bringing the families, other citizens, and partners together, indicating the crucial role of the broker [30]. Although creating the structural networks was mainly done by the broker via a top-down approach, the functionality of the network, e.g., the shared mission and the companionship that arose and the fun they had together as described by the families, happened as a bottom-up process in which the families and partners collaborated and were empowered [26]. However, it is important to realize that the broker remained vital as a back-up for the network connections to be maintained [31,32]. 

Traditionally, governments tend to take a more top-down controlling perspective, making families often ‘afraid’ to collaborate or ‘suspicious’ [33,34]. The close connection with the municipality in Vaals Meets also provided the opportunity for the government to change its governance style into collaborative governance. The municipality became ‘just another partner’ for the families to work together with. The families mostly appreciated the input from the policy maker, and now and then visiting meetings and being present at small-scale activities was valued. Key concepts, such as accessibility, transparency, and trust seemed to be enhanced during the study period [35]. This might have reduced the gap between policy and practice. The top-down perspective with which proposals and activities of the families were ‘judged’ about ‘what are the benefits?’ made way for a more balanced, constructive approach with the policy maker as one of the supporting partners empowering the families, while helping them in making their plans realistic. Making them meet in the ‘middle’.

### 4.1. Strengths and Limitations

A major strength of this study was the participatory design in which the researcher had the possibility to focus on an equal partnership with the families. This helped her to get more insight into the needs of the families, in addition to the needs already gathered during the photovoice study. Second, by retrospectively reconstructing all activities, key moments could be selected that directly or indirectly seemed to influence the partnership with the families. By creating the helicopter view using the timeline methodology, key lessons learned could be synthesized. Third, the inclusion of different data sources and the member checks with the broker, policy maker, and families during the study period were helpful in creating a complete overview of all activities and key lessons. Finally, although the number of families that took an active role in the partnership was relatively low, many more low-SES families were reached during the small-scale activities. 

One limitation is that we did not gather information about the number of families that might have been indirectly supported because of their partnership with the families in our project. Secondly, the partnership process was carried out with families, professionals, and other partners within only one municipality. The generalizability may be limited, since these partnerships are expected to be context specific. However, we tried to increase the generalizability by searching for and elaborating on the principles, functions, and key lessons to support future participatory approaches. Finally, we do realize that the RAF might seem to be a too basic tool, since it only includes four forms of capital. Though, we selected RAF because it really supported us to categorize what we learned from the partnership process and to stay close to the basic needs of the families found during the photovoice study. Besides, we also interpreted the four types of capital relatively broadly in order to ensure that we could capture the key lessons learned.

### 4.2. Recommendations for Future Research and Practice

In practice, many initiatives are undertaken in which partnering with the community is an important element. However, these local partnerships are often not evaluated, making the process behind successes and failures often a ‘gut feeling’. More participatory research is needed to better understand processes that underlie sustained partnerships in the area of community engagement [34].

For practice, we encourage professionals working with citizens to focus on making the step to partnership with the community. Communities should not only be involved, collaboration and empowerment should be the aim of the partnership [26]. While working together on topics that are important to the target group, opportunities will arise to enhance their situation and improve their overall health and well-being. Partnership in developing, implementing, and evaluating activities can restore power imbalances and support a mutual interest among all partners and therefore attain shared benefits.

## 5. Conclusions

This study showed the clear need for professionals to continuously and flexibly ‘navigate the middle’ between bottom-up and top-down approaches in creating equal partnerships with low-SES families. During the small-scale activities carried out together with and by the families in this study, we found six key lessons helpful in enhancing the partnership and finding this balance. The moment that the professionals were able to stop using professional work routines, became flexible, accommodated the talents of the families, and started doing, a shift in human, social, and political capital became evident. A supportive social network surrounding families, while maintaining professional back-up over time, was needed to enable the families to become more resilient and empowered. On a governmental level, the creation of an open, transparent, and involved communication with a focus on collaborative governance was seen as helpful in maintaining trust among the target group. In this study, the professionals were supported with finding an optimal partnership balance between the bottom-up input of the families’ talents that increased over time and the top-down input of the professionals’ expertise. Optimizing this balance seemed to reduce barriers for low-SES families, enabling them to participate. 

## Figures and Tables

**Table 1:** Key lessons learned for professionals working in community partnerships.

Key Lessons
Key Lesson 1: Stop using professional work routines, rather accommodate the talents of the target group who needed to start ‘doing’ and see activities. (Human Capital)
Key Lesson 2: Be flexible and focus directly on the preferred activities of the target group, while providing support and facilitation as an equal partner. (Human Capital)
Key Lesson 3: Work towards the creation of a supportive network surrounding the target group to share successes with and to fall back on. (Social Capital)
Key Lesson 4: Maintain professional back-up over time for the target group. (Social Capital)
Key Lesson 5: Work in accordance with the principles of collaborative governance to make the steering horizontal instead of top-down. (Political Capital)
Key Lesson 6: Create open, transparent, realistic and involved communication from the municipality to build and maintain trust among the target group. (Political Capital)
